# Prefrontal responses to negative emotional cognitive stimuli are associated with future suicide attempts in patients with mood disorders: a near-infrared spectroscopy study

**DOI:** 10.3389/fnimg.2026.1955513

**Published:** 2026-10-23

**Authors:** Hisashi Kamimura, Takahiro Matsuoka, Hiroshi Okai, Naoki Shimizu, Shu Harada, Koji Matsuo

**Affiliations:** 1 Department of Psychiatry, Faculty of Medicine, Saitama Medical University, Saitama, Japan; 2 Shinsayama Kaede Clinic, Saitama, Japan

**Keywords:** bipolar disorder, dorsolateral prefrontal cortex, major depressive disorder, mood disorder, near-infrared spectroscopy, suicide

## Abstract

Early identification of suicide risk remains a critical challenge in psychiatry. Longitudinal evidence linking baseline brain responses to subsequent suicide attempts remains limited. In this exploratory prospective follow-up study, 29 patients with major depressive disorder or bipolar disorder underwent near-infrared spectroscopy (NIRS) during an emotional Stroop task at baseline; 22 completed the 6-month follow-up and were included in the analysis, five of whom attempted suicide. Baseline oxygenated hemoglobin (Oxy-Hb) responses during the threat-word condition were compared between patients with and without suicide attempts using Mann–Whitney *U* tests. The main exploratory NIRS measure was the mean normalized Oxy-Hb response across 15–45 s (periods b + c) in the left dorsolateral prefrontal cortex (DLPFC); the corresponding left orbitofrontal cortex (OFC) measure was also examined. The follow-up attempt group showed higher baseline Oxy-Hb responses in the left DLPFC (*U* = 7.00, Holm-adjusted *p* = 0.022) and left OFC (*U* = 5.00, Holm-adjusted *p* = 0.020). Exploratory Spearman’s rank correlations showed that baseline Quick Inventory of Depressive Symptomatology scores were associated with the periods b + c NIRS measures in both regions, suggesting that baseline depressive symptom severity may have contributed to the observed group differences. These preliminary findings suggest that altered prefrontal hemodynamic responses during threat-related emotional interference may be associated with subsequent suicidal behavior. Larger prospective studies are required to replicate these findings and clarify their clinical relevance.

## 1 Introduction

Suicide is a significant public health concern worldwide. Every year, more than 720,000 people die by suicide, and suicide attempts are even more frequent (World Health Organization, 2025). In 2021, suicide was the third leading cause of death among people aged 15–29 years (World Health Organization, 2025). Mental disorders, particularly major depressive disorder and bipolar disorder, are strongly associated with suicidal thoughts and behaviors (Nordentoft et al., 2011). Furthermore, a history of suicide attempts is an important risk factor for subsequent suicidal behavior (Ribeiro et al., 2016). However, identifying clinical and neurobiological features associated with subsequent suicidal behavior remains challenging. Therefore, examining objective measures that may complement clinical assessments is an important research priority.

Neuroimaging studies have investigated the neural mechanisms underlying suicidal thoughts and behaviors. Schmaal et al. reviewed the available neuroimaging evidence and proposed a neurobiological model implicating brain regions involved in emotional regulation, cognitive control, decision-making, and reward processing in the development of suicidal behavior (Schmaal et al., 2020). Based on this framework, our previous cross-sectional near-infrared spectroscopy (NIRS) study examined prefrontal hemodynamic responses to a negative emotional cognitive task in relation to suicide-related behaviors in patients with mood disorders (Kamimura et al., 2022). That study suggested that responses in the dorsolateral prefrontal cortex (DLPFC) and orbitofrontal cortex (OFC) were associated with clinical and psychological characteristics, such as depression, hopelessness, and impulsivity, which contribute to the transition from suicidal ideation to suicidal behavior. Although previous neuroimaging studies have contributed to our understanding of the neurobiological mechanisms of suicide-related behaviors, most have been cross-sectional in design. Longitudinal studies examining whether baseline brain function differs according to subsequent suicidal behavior remain limited.

NIRS can be performed in a seated position with relatively few physical constraints and repeated with minimal burden. These features may be advantageous in patients with severe depressive symptoms or suicidal ideation who may have difficulty tolerating lengthy examinations (Koike et al., 2013). Although NIRS is limited to cortical hemodynamic changes in superficial regions, it may offer a practical approach for examining prefrontal responses relevant to suicidal behavior in psychiatric settings.

The aim of the present study was to compare baseline prefrontal hemodynamic responses during an emotional cognitive task between patients who did and did not attempt suicide during a 6-month follow-up period. We specifically focused on DLPFC and OFC oxygenated hemoglobin responses, which had been associated with suicidal behavior in our previous cross-sectional analysis (Kamimura et al., 2022). Because these regions were selected based on findings from the same study sample, the present analysis should be considered exploratory rather than confirmatory.

## 2 Methods

### 2.1 Study design and participants

This exploratory prospective follow-up study compared baseline prefrontal hemodynamic responses and clinical characteristics between patients with mood disorders who did and did not attempt suicide during a 6-month follow-up period. Participants were patients with major depressive disorder (MDD) or bipolar disorder (BD) who had participated in the previous cross-sectional NIRS study (Kamimura et al., 2022). Patients with evaluable baseline NIRS data who completed the 6-month follow-up assessment were included in the present analysis. Of the 29 patients who underwent baseline NIRS assessment, 22 completed the follow-up assessment and were included in the analysis.

Participants were recruited from inpatient and outpatient services at Saitama Medical University Hospital. Diagnoses were established by psychiatrists using DSM-5 clinical interviews and the Mini-International Neuropsychiatric Interview (Otsubo et al., 2005; American Psychiatric Association, 2013). Major exclusion criteria included personality disorder, neurological or organic brain disease, substance abuse or dependence, serious medical illness, left-handedness, and estimated intelligence quotient below 80. Further details were reported previously (Kamimura et al., 2022).

At baseline, psychiatric symptoms were assessed using the following measures: the 17-item Hamilton Depression Rating Scale (HAMD-17) (Hamilton, 1960) and the Quick Inventory of Depressive Symptomatology (QIDS) (Rush et al., 2003) for depressive symptoms; the Young Mania Rating Scale (YMRS) (Young et al., 1978) for manic symptoms; the Barratt Impulsivity Scale, 11th Edition (BIS-11) (Someya et al., 2001) for impulsivity; and the Beck Hopelessness Scale (BHS) (Beck et al., 1974) for hopelessness. The Columbia Suicide Severity Rating Scale (C-SSRS) (Posner et al., 2011) was used to evaluate suicide attempts during the follow-up period. At the 6-month follow-up, psychiatric symptoms were reassessed using the HAMD-17, QIDS, YMRS, BIS-11, and BHS. The C-SSRS was administered at the 6-month follow-up to determine whether participants had engaged in suicidal behavior during the follow-up period. Participants were categorized according to whether they made at least one actual suicide attempt during the 6-month follow-up period. Suicidal ideation, preparatory acts, aborted attempts, and interrupted attempts were not included in this definition.

Diagnostic subtype and current mood state were retrospectively extracted from medical records. Diagnostic subtype was categorized as MDD, bipolar I disorder (BD I), or bipolar II disorder (BD II). Current mood state at baseline and at the 6-month follow-up was categorized as remission, depressive episode, manic episode, hypomanic episode, or mixed state. Because no participants were classified as having a manic episode, hypomanic episode, or mixed state, these categories were combined for descriptive presentation in Table 1. Diagnostic subtype and mood-state characteristics were summarized descriptively because of the small sample size.

**Table 1:** Demographic, clinical, treatment, and behavioral characteristics of participants.

Characteristics	MDFA (*n* = 5)	MDNFA (*n* = 17)	Statistics	*p*-value
Age	49.0 ± 16.1	54.4 ± 13.6	*U* = 6.50	0.002
Sex (male/female)	1/4	7/10	–	0.613
Diagnosis (MDD/BD I/BD II)	2/1/2	14/1/2	–	–
Estimated IQ	102.7 ± 7.6	104.6 ± 11.5	*U* = 42.50	1.000
Current mood state at baseline, *n* (%)				
Remission state	1 (20.0)	10 (58.8)	–	–
Depressive episode	4 (80.0)	7 (41.2)	–	–
Manic or hypomanic episode or mixed state	0 (0.0)	0 (0.0)	–	–
Baseline clinical variables				
HAMD-17	13.0 ± 5.8	5.5 ± 4.7	*U* = 11.50	0.011
YMRS	1.4 ± 0.9	0.4 ± 0.6	*U* = 15.50	0.031
C-SSRS—Severity of suicidal ideation	4.2 ± 0.8	2.8 ± 2.2	*U* = 30.00	0.359
BIS total score	65.6 ± 13.9	62.8 ± 15.0	*U* = 36.50	0.649
BIS attentional impulsiveness	18.0 ± 7.6	16.1 ± 4.8	*U* = 35.00	0.595
BIS motor impulsiveness	24.8 ± 4.3	21.1 ± 5.7	*U* = 25.50	0.189
BIS non-planning impulsiveness	22.8 ± 4.1	25.7 ± 6.3	*U* = 31.00	0.401
QIDS	18.0 ± 2.9	10.6 ± 5.7	*U* = 10.50	0.009
BHS	16.4 ± 1.9	11.9 ± 5.3	*U* = 20.00	0.085
Current mood state during follow-up, *n* (%)				
Remission state	0 (0.0)	14 (82.4)	–	–
Depressive episode	5 (100.0)	3 (17.6)	–	–
Manic or hypomanic episode or mixed state	0 (0.0)	0 (0.0)	–	–
6-month follow-up variables				
HAMD-17	16.2 ± 6.6	3.5 ± 3.7	*U* = 4.50	0.001
YMRS	0.4 ± 0.5	0.3 ± 0.6	*U* = 36.50	0.649
C-SSRS—Severity of suicidal ideation	4.0 ± 1.0	0.4 ± 0.9	*U* = 1.00	< 0.001
QIDS	18.0 ± 6.5	7.1 ± 6.5 (*n* = 14)	*U* = 7.50	0.007
BHS	14.0 ± 5.2	7.4 ± 5.0 (*n* = 14)	*U* = 10.50	0.019
Medication at baseline				
Antidepressant, *n* (%)	4 (80.0)	15 (88.2)	–	–
Mood stabilizer, *n* (%)	3 (60.0)	5 (29.4)	–	–
Antipsychotic, *n* (%)	2 (40.0)	9 (52.9)	–	–
Medication during follow-up				
Antidepressant, *n* (%)	5 (100.0)	16 (94.1)	–	–
Mood stabilizer, *n* (%)	3 (60.0)	3 (17.6)	–	–
Antipsychotic, *n* (%)	3 (60.0)	9 (52.9)	–	–
Other treatment during follow-up				
Psychotherapy, *n* (%)	0 (0.0)	0 (0.0)		
Neuromodulation therapy (ECT/rTMS), *n* (%)	1 (20.0)	1 (5.9)		
Behavioral data				
Accuracy (%)	96.9 ± 3.5	97.7 ± 2.1	*U* = 39.00	0.820
Reaction time (ms)	862.3 ± 168.8	961.3 ± 210.7	*U* = 31.00	0.401

Continuous variables are presented as mean ± SD. MDFA, patients with mood disorders who attempted suicide during follow-up; MDNFA, patients with mood disorders who did not attempt suicide during follow-up; MDD, major depressive disorder; BD, bipolar disorder; HAMD-17, 17-item Hamilton Depression Rating Scale; YMRS, Young Mania Rating Scale; C-SSRS, Columbia Suicide Severity Rating Scale; BIS, Barratt Impulsiveness Scale; QIDS, Quick Inventory of Depressive Symptomatology; BHS, Beck Hopelessness Scale; ECT, electroconvulsive therapy; rTMS, repetitive transcranial magnetic stimulation. At 6-month follow-up, QIDS and BHS data were available for 14 participants in the MDNFA group. *p* values are unadjusted and are provided for descriptive purposes.

Medication use during the 6-month follow-up period was retrospectively extracted from medical records and summarized according to whether participants received at least one antidepressant, mood stabilizer, or antipsychotic during the follow-up period. Other major treatment interventions during follow-up, including psychotherapy and neuromodulation therapy [electroconvulsive therapy (ECT) or repetitive transcranial magnetic stimulation (rTMS)], were recorded.

This study was approved by the Institutional Review Board of the Saitama Medical University Hospital (IRB No. 19038.03) and conducted in accordance with the latest version of the Declaration of Helsinki. All participants were fully informed of the study’s purpose, both in writing and orally, and only those who provided written consent participated.

### 2.2 NIRS measurement and tasks

We measured the relative changes in oxygenated hemoglobin (Oxy-Hb) concentration during an emotional cognitive task using a 52-channel NIRS system (ETG-4100, Fujifilm Healthcare Corporation, Tokyo, Japan). The measurement probes were arranged in a 3 × 11 array with an inter-probe distance of 3 cm and were symmetrically positioned over the frontotemporal regions. The lowest row of measurement channels was aligned along the T3–Fpz–T4 line according to the international 10–20 electroencephalography system. The anatomical locations of the channels were estimated using Montreal Neurological Institute coordinates (Tsuzuki et al., 2007).

Relative changes in Oxy-Hb concentration were measured using near-infrared light at wavelengths of 695 and 830 nm based on the modified Beer–Lambert law and were expressed in millimolar-millimeters. The temporal resolution was 100 ms. Motion artifacts were corrected using a 5-s moving-average method, and channels containing substantial noise or artifacts were excluded using the algorithm described previously (Takizawa et al., 2014).

The emotional Stroop task developed by Matsubara et al. was used during NIRS measurement (Matsubara et al., 2014). The stimuli consisted of two-character Japanese kanji words categorized as “happy,” “sad,” “threat,” or “neutral.” Emotional words were used as target stimuli, whereas neutral words served as control stimuli. Each word was presented in red, blue, yellow, or green for 2 s using Presentation^®^ software (Neurobehavioral Systems, Berkeley, CA, United States). Participants were explicitly instructed to attend to the meaning of each word while pressing the button on the controller corresponding to the color in which the word was displayed as quickly as possible. Each emotional condition consisted of a 30 s pre-task neutral block, a 60 s emotional-word block containing 30 words, and a 60 s post-task neutral block containing 30 words. The happy, sad, and threat conditions were administered consecutively in one of six predefined orders, covering all possible arrangements of the three emotional conditions. The predefined orders were assigned sequentially in the order of participant examination to reduce potential order and fatigue effects. Further details have been reported previously (Matsubara et al., 2014; Kamimura et al., 2022). Guided by our previous cross-sectional NIRS findings in the same study sample, the present analysis focused on the threat condition (Kamimura et al., 2022). This focus was also supported by prior studies indicating that attentional processing of suicide-related and negative emotional stimuli is associated with suicidal thoughts and behaviors (Cha et al., 2010; Olié et al., 2015). To reduce the number of comparisons in this small exploratory sample, statistical analyses were restricted to the threat condition.

### 2.3 Data analysis

Behavioral data, including the percentage of correct responses and reaction times in the threat-word task, were analyzed using baseline data from the previous cross-sectional study. NIRS data were linearly corrected using the 10-s pre-task and final 5-s post-task control segments, and the fitted Oxy-Hb time series for each of the 52 channels was normalized separately for each participant by dividing it by the standard deviation calculated from the corresponding channel during the threat-word task.

As in previous studies, the channels for analysis were grouped into eight regions: left and right upper frontal, middle frontal, lower frontal, and OFC regions. Consistent with our previous study, the left upper frontal region was referred to as the DLPFC region because it included multiple channels estimated to correspond to the DLPFC (Kamimura et al., 2022). Only channels in the bottom three rows of the frontal region were used. To evaluate changes over time, the measurement period was divided into eight 15-s periods, designated periods a–h. The 60-s threat-word block was divided into period a (0–15 s), period b (15–30 s), period c (30–45 s), and period d (45–60 s), relative to the onset of the threat-word block. The subsequent 60-s post-task period was divided into periods e–h. The mean change in Oxy-Hb concentration was calculated for each period and region (Kamimura et al., 2022).

### 2.4 Statistical analysis

#### 2.4.1 Participant background and behavioral data

Participant background, clinical, and behavioral characteristics were summarized descriptively. For descriptive between-group comparisons, Mann–Whitney U tests were used for continuous variables and Fisher’s exact tests for categorical variables. The resulting *p* values were unadjusted and are reported for descriptive purposes rather than for confirmatory inference.

#### 2.4.2 NIRS data

NIRS analyses were based on nonparametric between-group comparisons. Based on our previous findings (Kamimura et al., 2022), we focused on Oxy-Hb responses during the 15–45 s period of the threat-word block, corresponding to periods b (15–30 s) and c (30–45 s), in the left DLPFC and left OFC regions. The main exploratory NIRS measure was defined as the mean Oxy-Hb response in the left DLPFC region across these two periods (b + c) and was compared between patients with and without suicide attempts during follow-up using a two-sided Mann–Whitney *U* test. The left DLPFC was selected as the main exploratory region because of its involvement in cognitive-control processes during emotional interference (Song et al., 2017) and because our previous analysis showed differences in this region (Kamimura et al., 2022). The corresponding left OFC measure across periods b and c was examined as an additional exploratory comparison. Period-specific values for periods b and c in the left DLPFC and left OFC regions were also compared between groups using Mann–Whitney *U* tests as exploratory analyses. Because these regions and periods were selected based on findings from the same study sample, these analyses were considered exploratory rather than confirmatory. To explore the potential influence of baseline mood symptom severity while limiting the number of comparisons, exploratory Spearman’s rank correlations were calculated between baseline HAMD-17, QIDS, and YMRS scores and the periods b + c summary measures in the left DLPFC and left OFC. To address the risk of false-positive findings due to multiple testing, Holm’s method was applied separately to two sets of related exploratory analyses: the six between-group NIRS comparisons reported in Table 2 and the six correlations between baseline mood symptom scores and the periods b + c NIRS measures reported in Supplementary Table S2. Both unadjusted and Holm-adjusted *p* values are reported. Statistical significance for these analyses was evaluated based on the Holm-adjusted *p* values, with a two-sided significance level of 0.05.

**Table 2:** NIRS responses during the threat-word task.

Region	Period	MDFA	MDNFA	*U*	Unadjusted *p*	Holm-adjusted *p*
Left DLPFC (superior frontal)	Period b + c	1.65 ± 1.12	−0.33 ± 0.96	7.00	0.005	0.022
Left OFC	Period b + c	1.56 ± 0.69	−0.44 ± 1.04	5.00	0.003	0.020
Left DLPFC (superior frontal)	Period b	1.58 ± 0.92	−0.35 ± 0.96	7.00	0.005	0.022
Left DLPFC (superior frontal)	Period c	1.71 ± 1.36	−0.32 ± 1.14	10.00	0.011	0.022
Left OFC	Period b	1.46 ± 0.64	−0.44 ± 1.02	6.00	0.004	0.021
Left OFC	Period c	1.66 ± 0.98	−0.44 ± 1.16	8.00	0.007	0.022

Oxy-Hb, oxygenated hemoglobin; DLPFC, dorsolateral prefrontal cortex; OFC, orbitofrontal cortex; MDFA, patients with mood disorders who attempted suicide during follow-up; MDNFA, patients with mood disorders who did not attempt suicide during follow-up. Values are presented as mean ± SD. Between-group comparisons were performed using two-sided Mann–Whitney U tests. Holm’s method was applied across the six between-group NIRS comparisons shown in the table. Both unadjusted and Holm-adjusted *p* values are reported. Periods b and c correspond to 15–30 s and 30–45 s after the onset of the threat-word block, respectively.

All statistical analyses were performed using SPSS Statistics for Windows (version 27.0; IBM Corp., Armonk, NY, United States).

## 3 Results

### 3.1 Participants and background data

At baseline, 29 participants were enrolled. Seven participants withdrew consent during follow-up, leaving 22 participants for the final analysis. No significant baseline differences were observed between completers and non-completers (Supplementary Table S1). Among the completers, five attempted suicide during follow-up. Participants were divided into two groups: patients with mood disorders who attempted suicide during follow-up (MDFA) and patients with mood disorders who did not attempt suicide during follow-up (MDNFA). Participant characteristics are summarized in Table 1. At baseline, the MDFA group was younger and had higher HAMD-17, YMRS, and QIDS scores than the MDNFA group. Four of five patients in the MDFA group and seven of 17 patients in the MDNFA group were in a depressive episode. Diagnostic subtype and mood-state characteristics are summarized descriptively in Table 1.

At the 6-month follow-up, HAMD-17, C-SSRS suicidal ideation severity, QIDS, and BHS scores were higher in the MDFA group than in the MDNFA group. All five patients in the MDFA group were in a depressive episode at follow-up, whereas 14 of 17 patients in the MDNFA group were in remission. Medication use and other treatment interventions during follow-up are summarized descriptively in Table 1.

### 3.2 NIRS data

NIRS analyses were based on nonparametric between-group comparisons. The MDFA group showed higher Oxy-Hb responses across periods b and c in the left DLPFC region than the MDNFA group (*U* = 7.00, Holm-adjuste*d p* = 0.022). The corresponding left OFC measure across periods b and c showed a similar between-group difference (*U* = 5.00, Holm-adjusted *p* = 0.020). Individual distributions of the periods b + c summary measures are shown in Figure 1. Detailed results for the main and additional exploratory NIRS measures are provided in Table 2.

**Figure 1:**
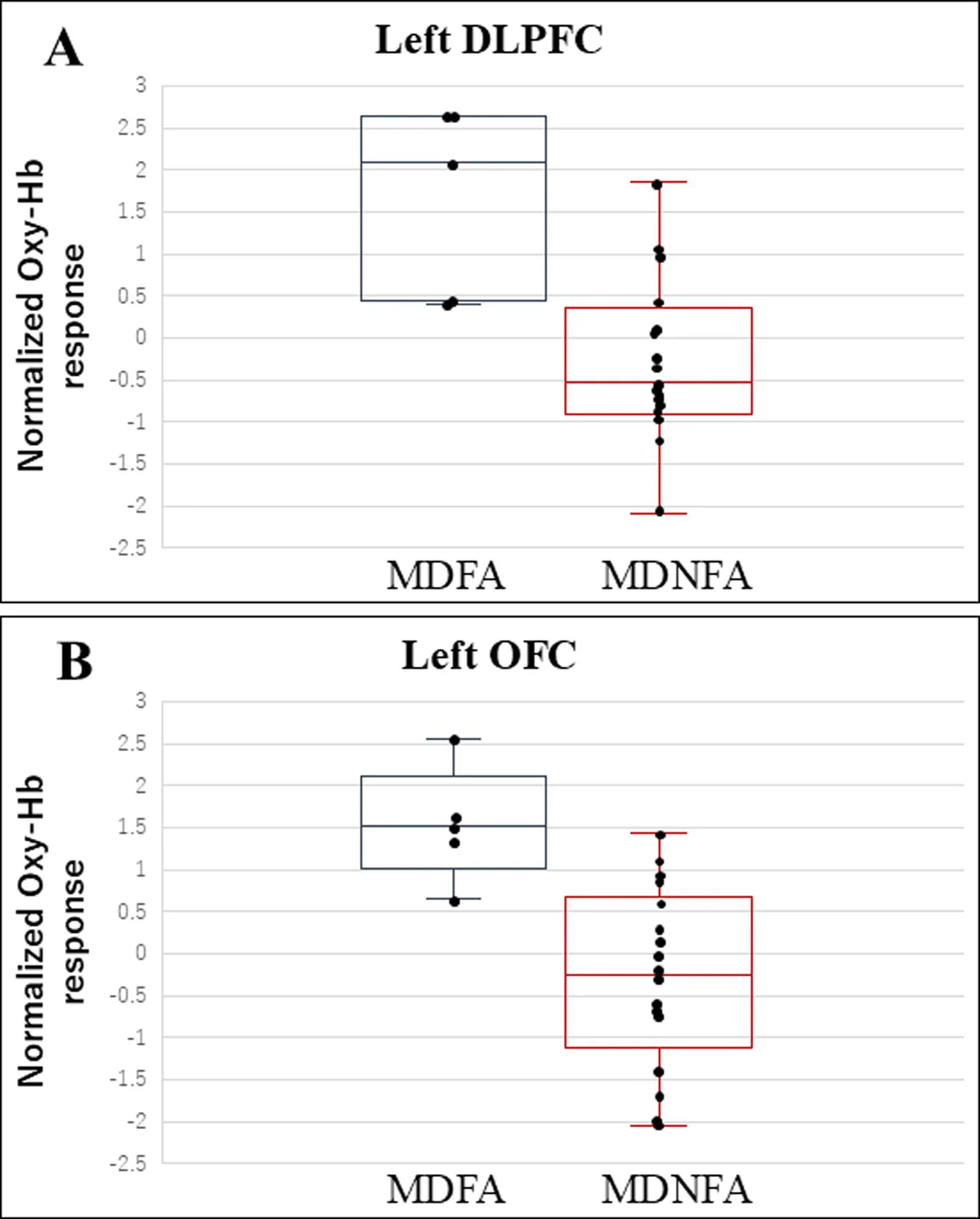
Baseline prefrontal NIRS responses during the threat-word task. Individual normalized Oxy-Hb responses averaged across periods b and c (15–45 s) are shown for the left DLPFC **(A)** and left OFC **(B)**. Each dot represents one participant. Between-group comparisons were performed using two-sided Mann–Whitney *U* tests. DLPFC, dorsolateral prefrontal cortex; OFC, orbitofrontal cortex; NIRS, near-infrared spectroscopy; Oxy-Hb, oxygenated hemoglobin; MDFA, patients with mood disorders who attempted suicide during follow-up; MDNFA, patients with mood disorders who did not attempt suicide during follow-up.

Additional period-specific analyses showed differences in the same direction for periods b and c in both the left DLPFC and left OFC regions (Table 2). Exploratory Spearman’s rank correlations were calculated between the periods b + c summary measures and baseline HAMD-17, QIDS, and YMRS scores. After Holm adjustment, QIDS scores remained significantly correlated with the periods b + c summary measures in both the left DLPFC and left OFC (Holm-adjusted *p* = 0.003 and 0.010, respectively). No other correlations remained significant after Holm adjustment. Detailed results are provided in Supplementary Table S2.

## 4 Discussion

To our knowledge, this is one of the first prospective NIRS studies to examine whether baseline prefrontal hemodynamic responses during an emotional cognitive task differ according to subsequent suicide attempts in patients with mood disorders. Our previous study suggested that the presence or absence of suicide attempts in patients with mood disorders was associated with differential responses in the DLPFC and OFC regions during negative emotional cognitive stimuli, and that these prefrontal responses may be related to psychological characteristics, such as impulsivity and hopelessness (Kamimura et al., 2022). The present prospective follow-up study extends these findings by showing that patients who attempted suicide during follow-up had greater baseline prefrontal Oxy-Hb responses during the threat-word task than those who did not attempt suicide.

The main finding was that the MDFA group showed greater mean Oxy-Hb responses across periods b and c in the left DLPFC region during the threat-word task. A similar between-group difference was observed in the corresponding left OFC measure. These findings suggest that altered prefrontal hemodynamic responses during threat-related emotional interference may be associated with subsequent suicidal behavior in patients with mood disorders. However, these findings should be interpreted as exploratory prospective associations, rather than evidence of predictive biomarkers or independent neural markers of suicide attempts.

Baseline clinical characteristics require careful interpretation. At baseline, the MDFA group was younger, had higher HAMD-17, YMRS, and QIDS scores, and more frequently met the criteria for a depressive episode than the MDNFA group. Although these between-group comparisons were descriptive and were not adjusted for multiple testing, exploratory correlation analyses showed that baseline QIDS scores remained significantly associated with the periods b + c NIRS responses in both the left DLPFC and left OFC after Holm adjustment. Taken together, these findings suggest that baseline depressive symptom severity may have contributed to the observed differences in prefrontal Oxy-Hb responses. Therefore, the influence of baseline depressive symptom severity on the observed prefrontal Oxy-Hb differences cannot be excluded. In addition, clinical scores measured at the 6-month follow-up should be interpreted separately from baseline variables. Higher HAMD-17, QIDS, BHS, and C-SSRS scores at follow-up may reflect clinical status after baseline and may have been influenced by suicidal behavior during the follow-up period, rather than representing predictors of suicide attempts.

The DLPFC plays an important role in complex decision-making by regulating emotions through its influence on emotion-related brain regions, such as the amygdala, and by exerting cognitive control in conflict situations (Mitchell, 2011). Dysfunction in these processes is considered relevant to the development of suicidal behavior. Consistent with this view, previous research has shown that individuals with a history of suicide attempts exhibit executive dysfunction and impaired neuropsychological functioning associated with the DLPFC (Raust et al., 2007).

Previous neuroimaging findings regarding prefrontal function in suicidal thoughts and behaviors have been inconsistent, and the direction of the observed alterations appears to depend on the brain region, task, and clinical population examined. Reduced prefrontal activation or fronto-limbic connectivity has been reported in some studies (Johnston et al., 2017; Brown et al., 2020). In contrast, increased recruitment of prefrontal regions has been observed during tasks involving negative emotion processing or emotionally influenced decision-making. Pan et al. reported greater activation in attentional control regions, including the left DLPFC, in adolescents with a history of suicide attempts during the processing of angry faces (Pan et al., 2013). Ji et al. (2021) investigated decision-making in untreated patients with depression and found higher DLPFC activation in those with a history of suicide attempts. Prefrontal alterations during decision-making with emotional feedback have also been reported in individuals with a history of suicide attempts (Alacreu-Crespo et al., 2020), and altered frontal-striatal control-related function has been associated with suicidal ideation and behavior in major mood disorders (Minzenberg et al., 2015). These findings suggest that altered functioning of the DLPFC and other prefrontal regions involved in emotional processing and decision-making may be associated with vulnerability to suicidal behavior. Greater DLPFC activation does not necessarily indicate more effective functioning. Increased prefrontal oxygenation during cognitively demanding tasks has been interpreted as reflecting greater recruitment of cognitive-control resources (Herrmann et al., 2003; Schroeter et al., 2004). In the present study, the greater DLPFC Oxy-Hb response in the MDFA group may therefore reflect altered recruitment of prefrontal resources when processing threatening information. However, it remains unclear whether this response represents adaptive compensation, less efficient processing requiring greater cognitive effort, or heightened sensitivity to threatening stimuli. Although behavioral accuracy and reaction time did not differ between groups, these measures cannot rule out differences in cognitive effort or the efficiency of emotional processing. Moreover, because baseline depressive symptom severity was greater in the MDFA group and QIDS scores were correlated with the NIRS responses, mood-state-related effects may also have contributed to the observed group difference. Medication effects may also have influenced task-related prefrontal hemodynamic responses. In addition, NIRS signals can be affected by systemic physiological and extracerebral hemodynamic changes that are not directly related to neuronal activity (Kirilina et al., 2012; Tachtsidis and Scholkmann, 2016). Therefore, the greater Oxy-Hb response observed in the MDFA group cannot be attributed solely to altered cortical neural processing.

The left OFC measure showed a between-group difference in the same direction as that observed in the left DLPFC. The OFC is involved in emotional evaluation, reward and punishment processing, and decision-making, processes that may be relevant to suicidal behavior (Kringelbach and Rolls, 2004). However, because the OFC analysis was additional and exploratory, the present findings do not support conclusions about the relative contributions of the DLPFC and OFC. Rather, the OFC result should be viewed as a preliminary finding suggesting that multiple prefrontal regions may be involved in altered responses to threat-related emotional stimuli.

The present sample included both patients with MDD and BD, which may involve partially distinct neural mechanisms related to suicidal behavior, including differences in prefrontal and fronto-limbic circuits (Johnston et al., 2017; Schmaal et al., 2020). Therefore, diagnostic heterogeneity should be considered when interpreting the present findings.

Overall, the present findings suggest that patients with mood disorders who subsequently attempted suicide may show altered prefrontal hemodynamic responses during threat-related emotional interference. This pattern is consistent with the possibility that prefrontal responses to negative emotional cognitive stimuli are related to vulnerability to suicidal behavior.

### 4.1 Limitations

Some limitations should be considered. First, the sample was small, and only five participants attempted suicide during the 6-month follow-up period. Therefore, multivariable modeling was not performed, and the effects of age, baseline depressive symptom severity, diagnosis, and other clinical factors could not be reliably separated. The MDFA group had higher mean HAMD-17 and QIDS scores at baseline than the MDNFA group in the descriptive comparisons, and baseline QIDS scores remained significantly associated with the NIRS responses after Holm adjustment. These findings raise the possibility that the greater prefrontal Oxy-Hb responses observed in the MDFA group were influenced, at least in part, by greater baseline depressive symptom severity. Thus, the observed prefrontal Oxy-Hb differences may partly reflect baseline clinical severity rather than suicide-attempt-specific neural alterations. Second, diagnostic heterogeneity between MDD and BD remains an important limitation because diagnosis-specific analyses were limited by the small sample size. Unmeasured confounding by socioeconomic status, family history, and childhood adversity also cannot be excluded because these factors were not assessed. Third, treatment during the follow-up period was not standardized. Differences in medication, psychotherapy, or other treatment changes may have influenced both clinical symptoms and suicidal behavior during follow-up. Because the present study was observational, the influence of treatment-related factors could not be excluded. Fourth, seven of the 29 baseline participants did not complete follow-up. Although no significant baseline differences were detected between completers and non-completers, the modest sample size limited the ability to detect such differences. Attrition-related selection bias therefore cannot be excluded, particularly if participants who discontinued follow-up differed in unmeasured clinical or psychosocial characteristics. Finally, NIRS measures superficial cortical hemodynamic changes and cannot directly assess deep limbic structures such as the amygdala (Yücel et al., 2021). In addition, NIRS signals may be influenced by systemic hemodynamic or physiological factors (Tachtsidis and Scholkmann, 2016). Therefore, the present findings should be interpreted as cortical hemodynamic correlates of emotional cognitive processing rather than direct evidence of whole-circuit dysfunction.

In conclusion, this exploratory prospective follow-up study showed that patients with mood disorders who attempted suicide during follow-up had greater baseline left DLPFC Oxy-Hb responses during the threat-word task than those who did not attempt suicide. A similar exploratory difference was observed in the left OFC. These preliminary findings suggest that prefrontal hemodynamic responses during threat-related emotional interference may be associated with subsequent suicidal behavior. However, given the small sample size, baseline clinical differences, diagnostic heterogeneity, and uncontrolled treatment factors, the findings should be interpreted cautiously; replication in larger prospective studies is required.

## Data Availability

The raw data supporting the conclusions of this article will be made available by the authors, without undue reservation.

## References

[ref1] Alacreu-Crespo A. Olié E. Le Bars E. Cyprien F. Deverdun J. Courtet P. (2020). Prefrontal activation in suicide attempters during decision making with emotional feedback. Transl. Psychiatry 10:313. doi: 10.1038/s41398-020-00995-z PMC7501865 32948747

[ref2] American Psychiatric Association (2013). Diagnostic and Statistical Manual of mental Disorders, Fifth edition. United States: American Psychiatric Publishing.

[ref3] Beck A. T. Weissman A. Lester D. Trexler L. (1974). The measurement of pessimism: the hopelessness scale. J. Consult. Clin. Psychol. 42, 861–865. doi: 10.1037/h0037562, 4436473

[ref4] Brown V. M. Wilson J. Hallquist M. N. Szanto K. Dombrovski A. Y. (2020). Ventromedial prefrontal value signals and functional connectivity during decision-making in suicidal behavior and impulsivity. Neuropsychopharmacology 45, 1034–1041. doi: 10.1038/s41386-020-0632-0, 32035425 PMC7162923

[ref5] Cha C. B. Najmi S. Park J. M. Finn C. T. Nock M. K. (2010). Attentional bias toward suicide-related stimuli predicts suicidal behavior. J. Abnorm. Psychol. 119, 616–622. doi: 10.1037/a0019710, 20677851 PMC2994414

[ref6] Hamilton M. (1960). A rating scale for depression. J. Neurol. Neurosurg. Psychiatry 23, 56–62. doi: 10.1136/jnnp.23.1.56, 14399272 PMC495331

[ref7] Herrmann M. J. Ehlis A. C. Fallgatter A. J. (2003). Prefrontal activation through task requirements of emotional induction measured with nirs. Biol. Psychol. 64, 255–263. doi: 10.1016/s0301-0511(03)00095-4, 14630406

[ref8] Ji X. Zhao J. Li H. Pizzagalli D. A. Law S. Lin P. . (2021). From motivation, decision-making to action: an fmri study on suicidal behavior in patients with major depressive disorder. J. Psychiatr. Res. 139, 14–24. doi: 10.1016/j.jpsychires.2021.05.007 34004553

[ref9] Johnston J. A. Y. Wang F. Liu J. Blond B. N. Wallace A. Liu J. . (2017). Multimodal neuroimaging of frontolimbic structure and function associated with suicide attempts in adolescents and young adults with bipolar disorder. Am. J. Psychiatry 174, 667–675. doi: 10.1176/appi.ajp.2016.15050652, 28135845 PMC5939580

[ref10] Kamimura H. Matsuoka T. Okai H. Shimizu N. Harada S. Matsuo K. (2022). The associations between suicide-related behaviors, prefrontal dysfunction in emotional cognition, and personality traits in mood disorders. Sci. Rep. 12:17377. doi: 10.1038/s41598-022-22345-3, 36253452 PMC9576702

[ref11] Kirilina E. Jelzow A. Heine A. Niessing M. Wabnitz H. Brühl R. . (2012). The physiological origin of task-evoked systemic artefacts in functional near infrared spectroscopy. NeuroImage 61, 70–81. doi: 10.1016/j.neuroimage.2012.02.074, 22426347 PMC3348501

[ref12] Koike S. Nishimura Y. Takizawa R. Yahata N. Kasai K. (2013). Near-infrared spectroscopy in schizophrenia: a possible biomarker for predicting clinical outcome and treatment response. Front. Psych. 4:145. doi: 10.3389/fpsyt.2013.00145, 24294205 PMC3827961

[ref13] Kringelbach M. L. Rolls E. T. (2004). The functional neuroanatomy of the human orbitofrontal cortex: evidence from neuroimaging and neuropsychology. Prog. Neurobiol. 72, 341–372. doi: 10.1016/j.pneurobio.2004.03.006, 15157726

[ref14] Matsubara T. Matsuo K. Nakashima M. Nakano M. Harada K. Watanuki T. . (2014). Prefrontal activation in response to emotional words in patients with bipolar disorder and major depressive disorder. NeuroImage 85, 489–497. doi: 10.1016/j.neuroimage.2013.04.098, 23643923

[ref15] Minzenberg M. J. Lesh T. A. Niendam T. A. Yoon J. H. Cheng Y. Rhoades R. N. . (2015). Control-related frontal-striatal function is associated with past suicidal ideation and behavior in patients with recent-onset psychotic major mood disorders. J. Affect. Disord. 188, 202–209. doi: 10.1016/j.jad.2015.08.049, 26363618

[ref16] Mitchell D. G. (2011). The nexus between decision making and emotion regulation: a review of convergent neurocognitive substrates. Behav. Brain Res. 217, 215–231. doi: 10.1016/j.bbr.2010.10.030, 21055420

[ref17] Nordentoft M. Mortensen P. B. Pedersen C. B. (2011). Absolute risk of suicide after first hospital contact in mental disorder. Arch. Gen. Psychiatry 68, 1058–1064. doi: 10.1001/archgenpsychiatry.2011.113, 21969462

[ref18] Olié E. Ding Y. Le Bars E. De Champfleur N. M. Mura T. Bonafé A. . (2015). Processing of decision-making and social threat in patients with history of suicidal attempt: a neuroimaging replication study. Psychiatry Res. 234, 369–377. doi: 10.1016/j.pscychresns.2015.09.020, 26483212

[ref19] Otsubo T. Tanaka K. Koda R. Shinoda J. Sano N. Tanaka S. . (2005). Reliability and validity of japanese version of the mini-international neuropsychiatric interview. Psychiatry Clin. Neurosci. 59, 517–526. doi: 10.1111/j.1440-1819.2005.01408.x, 16194252

[ref20] Pan L. A. Hassel S. Segreti A. M. Nau S. A. Brent D. A. Phillips M. L. (2013). Differential patterns of activity and functional connectivity in emotion processing neural circuitry to angry and happy faces in adolescents with and without suicide attempt. Psychol. Med. 43, 2129–2142. doi: 10.1017/s0033291712002966, 23298821

[ref21] Posner K. Brown G. K. Stanley B. Brent D. A. Yershova K. V. Oquendo M. A. . (2011). The columbia-suicide severity rating scale: initial validity and internal consistency findings from three multisite studies with adolescents and adults. Am. J. Psychiatry 168, 1266–1277. doi: 10.1176/appi.ajp.2011.10111704, 22193671 PMC3893686

[ref22] Raust A. Slama F. Mathieu F. Roy I. Chenu A. Koncke D. . (2007). Prefrontal cortex dysfunction in patients with suicidal behavior. Psychol. Med. 37, 411–419. doi: 10.1017/s0033291706009111, 17049103

[ref23] Ribeiro J. D. Franklin J. C. Fox K. R. Bentley K. H. Kleiman E. M. Chang B. P. . (2016). Self-injurious thoughts and behaviors as risk factors for future suicide ideation, attempts, and death: a meta-analysis of longitudinal studies. Psychol. Med. 46, 225–236. doi: 10.1017/s0033291715001804, 26370729 PMC4774896

[ref24] Rush A. J. Trivedi M. H. Ibrahim H. M. Carmody T. J. Arnow B. Klein D. N. . (2003). The 16-item quick inventory of depressive symptomatology (qids), clinician rating (qids-c), and self-report (qids-sr): a psychometric evaluation in patients with chronic major depression. Biol. Psychiatry 54, 573–583. doi: 10.1016/s0006-3223(02)01866-8, 12946886

[ref25] Schmaal L. Van Harmelen A. L. Chatzi V. Lippard E. T. C. Toenders Y. J. Averill L. A. . (2020). Imaging suicidal thoughts and behaviors: a comprehensive review of 2 decades of neuroimaging studies. Mol. Psychiatry 25, 408–427. doi: 10.1038/s41380-019-0587-x, 31787757 PMC6974434

[ref26] Schroeter M. L. Zysset S. Wahl M. Von Cramon D. Y. (2004). Prefrontal activation due to stroop interference increases during development--an event-related fNIRS study. NeuroImage 23, 1317–1325. doi: 10.1016/j.neuroimage.2004.08.001, 15589096

[ref27] Someya T. Sakado K. Seki T. Kojima M. Reist C. Tang S. W. . (2001). The japanese version of the barratt impulsiveness scale, 11th version (bis-11): its reliability and validity. Psychiatry Clin. Neurosci. 55, 111–114. doi: 10.1046/j.1440-1819.2001.00796.x, 11285088

[ref28] Song S. Zilverstand A. Song H. D'oleire Uquillas F. Wang Y. Xie C. . (2017). The influence of emotional interference on cognitive control: a meta-analysis of neuroimaging studies using the emotional stroop task. Sci. Rep. 7:2088. doi: 10.1038/s41598-017-02266-2, 28522823 PMC5437037

[ref29] Tachtsidis I. Scholkmann F. (2016). False positives and false negatives in functional near-infrared spectroscopy: issues, challenges, and the way forward. Neurophotonics 3:031405. doi: 10.1117/1.NPh.3.3.031405, 27054143 PMC4791590

[ref30] Takizawa R. Fukuda M. Kawasaki S. Kasai K. Mimura M. Pu S. . (2014). Neuroimaging-aided differential diagnosis of the depressive state. NeuroImage 85, 498–507. doi: 10.1016/j.neuroimage.2013.05.126, 23764293

[ref31] Tsuzuki D. Jurcak V. Singh A. K. Okamoto M. Watanabe E. Dan I. (2007). Virtual spatial registration of stand-alone fnirs data to mni space. NeuroImage 34, 1506–1518. doi: 10.1016/j.neuroimage.2006.10.043, 17207638

[ref32] World Health Organization (2025). Suicide Worldwide in 2021: Global Health Estimates. Geneva: World Health Organization.

[ref33] Young R. C. Biggs J. T. Ziegler V. E. Meyer D. A. (1978). A rating scale for mania: reliability, validity and sensitivity. Br. J. Psychiatry 133, 429–435. doi: 10.1192/bjp.133.5.429, 728692

[ref34] Yücel M. A. Lühmann A. V. Scholkmann F. Gervain J. Dan I. Ayaz H. . (2021). Best practices for fNIRS publications. Neurophotonics 8:012101. doi: 10.1117/1.NPh.8.1.012101, 33442557 PMC7793571

