# Supplementary material for: Prefrontal responses to negative emotional cognitive stimuli are associated with future suicide attempts in patients with mood disorders: a near-infrared spectroscopy study
Source: Front Neuroimaging. 2026 Oct 23;5:1955513. doi: 10.3389/fnimg.2026.1955513 (PMC13739502; doi:10.3389/fnimg.2026.1955513)
Supplement: Supplementary file 1 [file Table_1.DOCX]

**Supplementary Table S1. Baseline characteristics of completers and non-completers**

|  | Completers  (n = 22) | Non-completers  (n = 7) | Statistics | *p*-value |
| --- | --- | --- | --- | --- |
|  |  |  |  |  |
| Age | 49.7 ± 15.0 | 48.3 ± 15.5 | U = 72.50 | 0.823 |
| Sex (male/female) | 8/14 | 2/5 | – | 1.000 |
| Diagnosis (MDD/BD I/BD II) | 16/2/4 | 6/0/1 | – | – |
| Estimated IQ | 104.2 ± 10.6 | 101.9 ± 15.1 | U = 72.00 | 0.823 |
| **Baseline clinical variables** |  |  |  |  |
| HAMD-17 | 7.2 ± 5.8 | 8.3 ± 4.8 | U = 64.00 | 0.533 |
| YMRS | 0.6 ± 0.8 | 1.1 ± 2.2 | U = 75.00 | 0.940 |
| C-SSRS - Severity of suicidal ideation | 3.1 ± 2.0 | 2.1 ± 2.1 | U = 52.50 | 0.217 |
| BIS total score | 63.5 ± 14.5 | 63.4 ± 5.7 | U = 70.00 | 0.746 |
| QIDS | 12.3 ± 6.1 | 11.4 ± 6.0 | U = 69.00 | 0.709 |
| BHS | 12.9 ± 5.1 | 11.0 ± 5.0 | U = 57.50 | 0.328 |
| **Medication at baseline** |  |  |  |  |
| Antidepressant, *n* (%) | 19 (86.4) | 6 (85.7) | – |  |
| Mood stabilizer, *n* (%) | 8 (36.4) | 2 (28.6) | – |  |
| Antipsychotic, *n* (%) | 11 (50.0) | 4 (57.1) | – |  |
| **Behavioral data** |  |  |  |  |
| Accuracy (%) | 97.5 ± 2.5 | 97.6 ± 2.4 | U = 72.50 | 0.823 |
| Reaction time (ms) | 938.8 ± 202.6 | 891.9 ± 175.1 | U = 68.00 | 0.672 |

Continuous variables are presented as mean ± SD.

MDD, major depressive disorder; BD, bipolar disorder; HAMD-17, 17-item Hamilton Depression Rating Scale; YMRS, Young Mania Rating Scale; C-SSRS, Columbia Suicide Severity Rating Scale; BIS, Barratt Impulsiveness Scale; QIDS, Quick Inventory of Depressive Symptomatology; BHS, Beck Hopelessness Scale.

P values are unadjusted and are provided for descriptive purposes.
