# Supplementary material for: Prefrontal responses to negative emotional cognitive stimuli are associated with future suicide attempts in patients with mood disorders: a near-infrared spectroscopy study
Source: Front Neuroimaging. 2026 Oct 23;5:1955513. doi: 10.3389/fnimg.2026.1955513 (PMC13739502; doi:10.3389/fnimg.2026.1955513)
Supplement: Supplementary file 2 [file Table_2.DOCX]

**Supplementary Table S2.** Spearman’s rank correlations between baseline mood symptom scores and NIRS responses

| Baseline symptom measure | *ρ* | Unadjusted  *p* | Holm-adjusted  *p* |
| --- | --- | --- | --- |
| **Left DLPFC, periods b+c mean** |  |  |  |
| HAMD-17 | 0.345 | 0.067 | 0.201 |
| QIDS | 0.603 | 0.001 | 0.003 |
| YMRS | 0.300 | 0.114 | 0.228 |
| **Left OFC, periods b+c mean** |  |  |  |
| HAMD-17 | 0.254 | 0.183 | 0.228 |
| QIDS | 0.551 | 0.002 | 0.010 |
| YMRS | 0.389 | 0.037 | 0.149 |

ρ, Spearman’s rank correlation coefficient; HAMD-17, 17-item Hamilton Depression Rating Scale; QIDS, Quick Inventory of Depressive Symptomatology; YMRS, Young Mania Rating Scale; DLPFC, dorsolateral prefrontal cortex; OFC, orbitofrontal cortex; NIRS, near-infrared spectroscopy; Oxy-Hb, oxygenated hemoglobin. Periods b+c mean represents the mean normalized Oxy-Hb response across periods b and c (15–45 s) of the threat-word block.

Holm’s method was applied across the six correlation analyses shown in the table. Both unadjusted and Holm-adjusted *p* values are reported.
